# Designing the Well-Being of Romanians by Achieving Mental Health with Digital Methods and Public Health Promotion

**DOI:** 10.3390/ijerph19137868

**Published:** 2022-06-27

**Authors:** Gabriel Brătucu, Andra Ioana Maria Tudor, Adriana Veronica Litră, Eliza Nichifor, Ioana Bianca Chițu, Tamara-Oana Brătucu

**Affiliations:** 1 Faculty of Economic Sciences and Business Administration, Transilvania University of Brașov, Colina Universității Street No. 1, Building A, 500068 Brașov, Romania; gabriel.bratucu@unitbv.ro (G.B.); andra.tudor@unitbv.ro (A.I.M.T.); eliza.nichifor@unitbv.ro (E.N.); ioana.chitu@unitbv.ro (I.B.C.); 2 The School Center for Inclusive Education Brasov, 125 Bd. 13 Decembrie, 500164 Brașov, Romania; tamara.bratucu@cseibv.ro

**Keywords:** mental health promotion, well-being, depression prevention, digital health, public mental health policies

## Abstract

Taking care of mental health is a state of mind. Amid the challenges of the current context, mental health has become one of the problems with the greatest impact on citizens and the evolution of any economy. Due to the COVID-19 pandemic, people have become more anxious, solitary, preoccupied with themselves, and depressed because their entire universe has changed, by restricting their social and professional life; the increase in concern caused by a possible illness of them or those close to them made to isolate themselves. Two qualitative (group and in-depth interviews) and one survey-based quantitative research were carried out, which allowed the quantification of the opinions, perceptions, and attitudes of Romanians regarding the effectiveness of policies for the prevention and treatment of depression. Quantitative research revealed that most of the subjects had never participated in a mental health assessment, and a quarter of them had visited a mental health specialist more than two years ago. Based on the results, proposals were elaborated, which have been addressed both to the specialists from the Ministry of Health and to those from the academic environment, that may have an impact on the elaboration of some public mental health programs.

## 1. Introduction

Mental health is a state of the successful performance of the mental function, resulting in productive activities, fulfilling relationships with other people, and the ability to adapt to change and cope with challenges. Mental health is essential to personal well-being, family and interpersonal relationships, and the ability to contribute to the community or society [1].

Mental disorders are health conditions that are characterized by alterations in thinking, mood, and/or behavior that are associated with distress and/or impaired functioning [1]. Mental disorders include depression, bipolar disorder, schizophrenia, and other psychoses, dementia, and developmental disorders, including autism [2].

The article aimed at quantifying the opinions, perceptions, and attitudes of Romanians on the effectiveness of policies to prevent and treat depression. To this end, the authors conducted a series of research, as follows: (1) a qualitative research of group interview type, which had the following objectives: (O_1_) identification of information methods on mental illness; (O_2_) perception of Romanians towards prevention and treatment of depression and anxiety; (O_3_) determination of knowledge regarding the methods to prevent depression; (O_4_) evaluation of awareness regarding the mental illness prevention campaigns. (2) an in-depth interview-type qualitative research whose objectives were: (O_1_) identifying the perceptions of the interviewees regarding the impact of COVID-19 on the individual (mental health, consumption behavior, etc.), (O_2_) determining the methods to stimulate mental health concerns in the context of the COVID-19 pandemic, (O_3_) quantification of views on ways to respond to mental health needs as a result of the pandemic experience; (3) a quantitative survey research whose objectives have been formulated following the interpretation of the results obtained in the qualitative research: (O_1_) measuring the importance given to mental health by Romanians, (O_2_) identifying the factors that stimulate mental health care among Romanians, (O_3_) determining the frequency of consumption of mental health services, (O_4_) quantifying the degree of information on the symptoms of depression, and (O_5_) quantification of the annual budget allocated by Romanian adults for the prevention of mental illness.

Following quantitative research, the percentage of respondents who stated that the symptoms of depression are known is 67.1%. However, 67.9% of the subjects stated that they had never participated in a specialist assessment for their mental health, and 27.5% said that the last visit to a specialist for mental health assessments took place more than two years ago.

### 1.1. Literature Review

In the opinion of the specialists, it is difficult to define exactly the meaning of the concept of good mental health; however, core domains that may capture the essence of it encompass (i) mental health literacy, (ii) attitude towards mental disorders, (iii) self-perceptions and values, (iv) cognitive skills, (v) academic/occupational performance, (vi) emotions, (vii) behaviors, (viii) self-management strategies, (ix) social skills, (x) family and significant relationships (xi) physical health, (xii) sexual health, (xiii) meaning of life, and (xiv) quality of life [3].

According to GHDx data [4], in 2019, the prevalence of mental disorders globally was 970 million people, of which approximately 67 million people were from Western Europe and 13 million people were from the Central European states, and 2.2 million people were from Romania.

Calculated as a percentage (proportion of total cases of a particular cause relative to cases from all causes), mental disorders in Romania represented 11.89%, of which depressive disorders amounted to 3.51% and anxiety disorders to 3.86% (Table 1).

As a prevalence rate per 100,000 population, Romania had in 2019 a rate of 11,423 cases of mental disorders, of which 3368 were depressive disorders and 3711 were anxiety disorders; these values are close to the Central European average and lower than the values recorded globally but well below than those recorded for Western Europe.

In the literature dedicated to the means of prevention/assistance/information in the field of mental disorders, a special place is reserved for the concept of positive mental health. This is a result of the well-being of an individual on multiple levels (*emotional well-being*—the presence of positive affect and satisfaction with life; *social well-being*—social functioning, integration into society, acceptance, involvement, and contribution; *psychological well-being*—intrapersonal and interpersonal functioning, self-acceptance, and personal development) [5].

The concepts of well-being and positive mental health do not overlap, as a high level of any of them can occur in the presence of a low level of the other. However, the connection between them can be summarized by the fact that positive mental health generally contributes to a fulfilled life, which includes, but is not limited, to well-being [6].

High levels of positive mental health protect individuals from mental disorders, helping them recover from mental illness and stay mentally healthy [7]. Positive mental health can mediate the relationships between mental disorders and health outcomes, acting as an underlying mechanism to improve overall health and quality of life in individuals with mental disorders [8].

In developing mental disorders, symptoms commonly include depression, anxiety, or stress. People experiencing mental disorders score lower on measures of subjective well-being [9]. Subjective well-being is not a uniformly defined concept, but it encompasses a cognitive component evaluating satisfaction with life and an affective component involving positive affect.

Linking mental health to the economic and development degree, many studies confirm a vulnerability paradox: a positive connection at the individual level, and on the contrary, lower vulnerability at the country level is accompanied by a higher prevalence of mental disorders in national populations [10]. Starting from the observation that cross-national studies confirm higher mental disorder prevalence in high-income countries, Scott, Zhang, Chardoul et al.’s [11] research provided consistent results with reduced prevalence of mental disorders in other low-income countries.

Stigma is a recognized issue related to people facing mental disorders [12]. In the case of mental disorders, stigma is manifested in two forms [13]: “public stigma”—when other people perceive a mentally ill person as socially undesirable, and ”self-stigma”—as a result of internalizing perceived prejudices, stigmatized individuals develop negative views about themselves. Stigma exerts negative effects in two ways, impeding treatment participation: it diminishes self-esteem and robs people of social opportunities [14].

Both internalized (self) stigma and public stigma proved a negative causal link with help-seeking [15]. For fear of being labeled with mental illness, many people avoid seeking assistance or fully participating in care [16]. Starting from the finding that less than one-third of individuals with mental health problems receive treatment, Vidourek and Burbage [17] found that a majority of college students consider stigma as being a barrier to mental health treatment.

An important part of preconceptions about mental illness is shared. Latalova, Kamaradova, Prasko et al. [13] found that gender and race are related to stigma. Among depressed patients, males and African Americans have higher levels of self-stigma than females and Caucasians. Conformity to dominant masculine gender norms (men are strong) is one of the reasons why men feel compelled to cope with depression without seeking professional help. A similar conclusion was drawn by Mackenzie, Visperas, Ogrodniczuk et al. [18], finding that men are more likely to take their own lives but less likely to seek professional help when they are depressed, especially as they age. They also found that public stigma was the highest in younger adults (especially young men), while self-stigma appeared to be independent of sex, but higher in younger adults.

In different crisis contexts, regardless of their origin—economic, war, or pandemics— individuals face altered well-being, and this might be reflected in multiple forms. The incidence of these outcomes is closely related to the level of resilience of these four people. Resilience is the individual capacity to adapt to adverse life events, being able to recover and return to a positive condition [19]. Resilience refers to the ability to cope with difficult, stressful, and traumatic situations and to return to the previous healthy condition, avoiding the occurrence of significant dysfunctions. The higher the resilience, the lower the vulnerability to developing an illness [20,21,22].

The main goal of mental health awareness and knowledge is to increase the willingness in seeking assistance and treatment when needed, as earlier interventions might mitigate the burden of mental disorders. Due to the important relationship to mental illness, resilience has the potential to be used as a defensive measure toward specific mental health conditions such as depression and anxiety. Resilience has become a central concept in the field of emergency and disaster management. Interventions based on a combination of cognitive behavioral therapy and mindfulness training appear to have a positive impact on individual resilience [23,24,25].

Resilience training has been found to have a positive impact on personal resilience (“purpose in life, self-esteem, interpersonal relationship, job satisfaction”) [26], occupational stress [27], and personal wellbeing [28] and helps people handle daily stress [29,30].

Particularly, during times of difficulty or crises, mental health outcomes differ among individuals, and psychological resilience is expected to play an important role in explaining this variance [31]. In the coronavirus pandemic, the stress emerging from health-related risks, uncertainty, and quarantine measures may trigger or exacerbate pre-existing conditions related to mental disorders [32].

### 1.2. COVID-19 and Mental Health

The coronavirus pandemic has been declared by the World Health Organization a public health emergency of international interest [33]. The pandemic context has had a devastating effect not only on supply chains, economic life, travel, and global health systems but especially has had and still has a negative effect on the general health of the population [34]. The pandemic is likely to have made society aware of mental health issues and how they should be managed [35]. On the other hand, it is this pandemic that has revealed in some countries the insufficient preparation of health systems [36].

COVID-19 has imposed barriers that have damaged the routine and behavior of all individuals, be they children, adolescents [37] adults, or the elderly, as these barriers can affect the mental health of those mentioned above, which requires the development of specific intervention plans based on the needs of each vulnerable group [38,39]. A priority is considered to be the assessment of these disorders in those who have survived the disease [40].

The impact of COVID-19 on the business environment has led to job uncertainties and a socio-economic crisis among the population [41]. A large part of the employees in the companies encountered difficulties in accessing the workplace, suffered reductions in salaries, switched to the telework program, or, in the worst case, were fired [42,43].

Studies conducted on health care workers have found that their mental health was more affected than that of workers in another fields, and this is associated with emotional well-being [44,45]. Another group mentioned by the studies is that of children and young people [46], especially those with special needs [47], because the education system has also been affected and the educational institutions being closed; thus, the children’s educational routine as well as the social life were affected [48].

People who follow social distancing measures experience loneliness, which most often results in anxiety, anger, misperceptions about life [48], post-traumatic stress symptoms, and depression [49]. Consequently, the pandemic has led to an increase in the level of anxiety and fear related to the management and spread of this virus among the population [50,51,52,53]. Moreover, the increased contagion of the virus has, in turn, created an increase in confusion, panic, and fears among the population. These, together with the measures of social distancing imposed at the level of each state, have affected the mental well-being of individuals [49,54], especially for people with such pre-existing problems [55], which implies an even greater need for the involvement of public authorities (especially those in the health field) in terms of mental health issues. The fact that nowadays society is so interconnected entails both an increase in the vulnerability of the population to this virus but also in facilitating the exchange of information so as to create the necessary infrastructure to improve mental health services [55].

On the above-mentioned aspects can be added the fact that myths, inaccurate information, and the phenomenon of “fake news” about COVID-19 have spread extremely rapidly in both traditional media and social media, with a negative impact on the mental health of individuals [43,56,57]. Fears about the transmission of the virus, the illness itself, or the experience of the disease lived by a family member or close acquaintance have an impact on the mental functions of human nature. At the same time, the psychological fear of a condition can generate anxiety and depressive symptoms even more than the disease itself [58]. Therefore, the problems related to the mental health of the individual can intensify in epidemic or pandemic contexts.

Studies show that the impact of the virus will be significant and lasting, especially in countries that do not have sufficient resources to invest in public mental health policies. Additionally, they should include a wide variety of experiences and actions included by these diseases, determined by the social context [59,60].

Given these considerations, promoting the resilience of mental health and addressing issues related to it should be a priority of public health policies [39]. Communication should be based on compassion (compassion for oneself and others) [61] and positive psychology, given the inverse relationship between happiness, resilience, and fear [62].

In Romania, especially during the lockdown period, there was no clear strategy for sustainable communication at the national level with clear procedures, the communication being often made by unprepared people [63]. Information through the media was not completely correct and useful but rather “exaggerated, confusing, false”, leading to anxiety and tension [64]. Post lockdown, communication has improved [63], the Romanian Ministry of Health also implementing a free telephone line for psycho-emotional support for people affected by the COVID-19 pandemic [65].

## 2. Materials and Methods

The first research was qualitative research, aiming at quantifying the attitudes and knowledge of Romanian adults regarding the prevention and treatment of mild mental illnesses. The research involved conducting a group interview in five locations in the most important urban centers in Romania. Two groups were formed for each location, each group having eight participants. Thus, 80 adults from Romania, aged between 18–60, were interviewed. In both qualitative surveys conducted, the research population targeted the same age range because the authors did not want to include people over 60 years of age. It was taken into account that in Romania, many employees retire early at the age of 60, although officially the retirement ages are 62 for women and 65 for men. The decision was made in order not to discriminate against the subjects included in the samples, taking into account the legal retirement age limit. The members of the sample were selected by applying a recruitment questionnaire (distributed in 5 psychology offices, 5 psychiatric centers, and 10 family medical offices). The respondents to the recruitment questionnaire who met the selection criteria were subsequently contacted by telephone. The group interview was initiated by the presentation of the rules of the discussion, the introduction of the participants, and a short period of accommodation of the participants. The preamble of the group discussions in the qualitative marketing research undertaken had an allotted time of about 5 min. Following the preamble, discussions were directed to the topics of the interview guide, which lasted approximately 60 min. Qualitative marketing research was conducted between May and June 2021. Discussions within the ten focus groups were recorded, being analyzed in detail afterward. The method of analysis used in this qualitative research was the content analysis, and the data obtained from each group were analyzed both vertically and horizontally.

The second stage of the research aimed at conducting a semi-structured in-depth individual interview. Thus, we started from an interview guide on the topic regarding the opinions, perceptions, attitudes, and behaviors of Romanians regarding mental health, digital tools, and their use in the context of the COVID-19 pandemic. The research was based on open-ended questions addressed to adults in Romania, former COVID-19 patients, or direct contacts of former COVID-19 patients, and involved the interview of 36 adults in Romania, 18 women and 18 men, aged between 18–60 years, the duration of each interview being of 60 min. This qualitative research took place in September 2021, with the aim of determining detailed information on the behavior of health care users, the impact of the pandemic on mental health and well-being, and ways to adapt and respond to mental health needs in this context. Due to the pandemic conditions, the recruitment process specific to the qualitative research has not been done in the classic way by calling the subjects by telephone. Under the given conditions, the participants in this research were recruited with the help of social networks, from groups whose interests focused on events, actions, activities, and interactions on topics focused on the pandemic, health in general and mental health, etc.

The interview guide was structured into four topics:

A. General knowledge and perceptions about COVID-19.
B. Health concerns and the impact of COVID-19 on mental health.
C. Methods of communication, mental health information, and adaptation to the new reality.
D. Perspectives and recommendations in addressing mental health needs.

The techniques applied in the construction of the interview guide, for the collection of qualitative data are:

- Open-ended question technique (all topics of the interview guide);
- The technique of situational/hypothetical questions (topics C and D in the interview guide).

Based on the obtained results, the qualitative research conducted by the authors to the quantitative survey-based research, the data being collected through a questionnaire, administered online, using the Computer-Assisted Web Interviewing (CAWI) method. Data collection was conducted between October 2021 and January 2022. The questionnaire included 29 questions, of which 23 questions were regarding the opinions, attitudes, and behaviors of Romanian adults on the use of digital tools to prevent depression, and 6 questions were to identify them. 514 subjects (women and men over the age of 18) were interviewed, and a non-random sampling method was chosen, using a combined method of the snowball, the survey based on volunteering, and the one based on rational selection criteria. After collecting the data, the database with the completed questionnaires was downloaded, then the answers were coded and processed using the Statistical Package for Social Sciences program (SPSS Statistics 25). A total of 522 questionnaires were completed, of which 8 were excluded from the marketing analysis, being invalidated by the researcher (inconsistency in answers, incomplete answers, etc.). This led to a sample of 514 people.

## 3. Results

In the following part, the results obtained in the order of performing the three research will be detailed.

### 3.1. The Results of the Focus Group

The study revealed that 30% of the subjects consider that, most often, Romanians use the Internet to self-assess their mental health. Most subjects say that people are late to seek medical advice, either because of the stigma associated with the terms “psychiatrist” or “psychologist”, or because of ignorance, lack of information, or lack of access to health care. Many of the subjects considered friends and family to be their first “place of refuge”, where people go to analyze their condition, because ”we are always looking for confirmations from the close ones if it seems to us that something is going or not going in a certain direction. It’s in our nature.” Some subjects believe that people often go to assess their mental health to a psychologist and not a psychiatrist, and a small part of the subjects include family doctors as the first choice when people go to assess their mental health, due to the role that the family doctor has in issuing referrals to specialized medical units.

60% of the participants state that they have not organized information campaigns or complex actions on depression. The subjects mention that in the units, the patients who use their services benefit from information on the symptoms, prevention methods, risks, etc., but that the actions in this direction are neither complex nor sustained within the units. At the same time, some medical units develop patient guidelines for depression and treat the subject through online information actions on their website/blog or even integrate mental health into private healthcare services. However, the subjects cite the lack of resources and support from the state as reasons for the small and far-reaching actions for information on the prevention, detection, and treatment of depression.

Most of the subjects consider that there is a real need for greater involvement of the state through its institutions (Ministry of Health, Ministry of Education, etc.) in organizing such campaigns. At the same time, the subjects consider the involvement of all parties (public and private system) essential, being of the opinion that the organization of such campaigns should not fall exclusively to the NGOs, as is usually the case. In the view of the subjects, for the organization of campaigns for the prevention or screening of depression, it should be close collaboration and accountability of each party involved: “Let the public institutions be responsible, let the medical units be responsible, and let all the rest of us be responsible for our mental health.”

Regarding campaigns on mental and behavioral disorders in collaboration with public institutions, 90% of the research subjects stated that they did not carry out such campaigns to prevent or detect depression in the early stages, in collaboration with state institutions. Only one subject mention such an action, carried out in the past: “(…) It was one, a few years ago. An action, I wouldn’t call it a campaign”.

When asked what are the weaknesses of the Romanian health system in this field, the answers focused on the same negative aspects of the Romanian health system in the field of mental health, namely: lack of staff, lack of infrastructure, lack of clear protocols, untrained family doctors in terms of mental and behavioral disorders, lack of national campaigns on these diseases, lack of investment in the field, lack of prevention services, corruption, lack of health education, overcrowded hospitals, excessive bureaucracy, disinterest, and weak communication.

Many subjects believe that family doctors and also school doctors should be the basic components of such campaigns. Beyond the educational component, which is primordial, the family doctor is the main source of medical information in rural areas, so that, in the view of research subjects, they should be points of support in the efficient running of prevention campaigns. 70% of the research subjects consider that, in the campaigns for the prevention of mental and behavioral disorders, the online environment is important in disseminating messages (through mobile applications, websites, social networks, etc.), especially for the young segment of the population. The subjects consider that the way the message is transmitted should differ according to the age segments and the residence environment. Most subjects believe that the first step in successful campaigns in this area should be to try to eliminate the stigmatization of mental and behavioral disorders through all existing means of communication.

Regarding early depression screening campaigns, the subjects believe that the approach and means of communication should differ according to the age of the target population. Thus, most subjects believe that the promotion of these campaigns should be done online, through means such as social media, video content platforms, blogs, specialized sites, mobile applications, and forums for the youth segment, while for adults and the elderly, the doctor and messages transmitted through traditional media would be more appropriate. Some subjects believe that free on-the-job assessments or free state-funded diagnostic campaigns would be desirable for depression screening.

The most frequent information methods used in the promotion/communication actions on the medical services mentioned by the subjects refer to face-to-face communication, blog, social networks, websites, outdoor banners, family doctors’ networks, specialized forums, and flyers.

Some of the subjects are reluctant not about using technology in communication but rather due to the lack of health education, which could lead to misunderstanding the messages transmitted through the tools of technology: “(…) It has a double meaning. On the one hand, it is difficult for us to use technology when not everyone proves the necessary skills, then there is also the education part (that in vain you send messages quickly if the person to whom you send the message does not know how to read, does not know how to discern subjects.” Subjects are aware of the advantages of using technology in health communication: data and information storage, faster feedback, lower costs, speed in disseminating information, etc. The usefulness of technology in communication, however, “(…) depends only on what we want to communicate through technology and to whom.”

The subjects generally believe that at the level of the population, the effects of information campaigns on depression have positive effects only if they are well built, if they will be supported and carried out in the long term, and are in the context of prioritization of investments in prevention and health education. At the same time, for the subjects, “(…) information campaigns can have an essential role and tailor-made effects, as long as the messages are constructed and transmitted in the language of the person to whom they are addressed. Otherwise, they are just other actions started just for the sake of reporting well”, which reflects the fact that the subjects consider it appropriate to change the approach of campaigns and diversify the means of information communication to generate the desired effects at the population level.

Regarding the reasons why Romanians would decide to go for medical check-ups, to assess the level of mental health, the main reasons listed by the research subjects refer to social factors—the urge of the family and friends, accessibility of medical services, regular information campaigns, improving health education, the power of example, free mental health assessment programs, settlements or discounts offered, but also to improve the quality of medical services in this area.

More than half of the interviewed subjects believe that factors such as fear, shame, or stigma associated with the field dealing with mental illness and behavior contribute to the fact that Romanians ignore the assessment of mental health with the help of specialists. Moreover, in the view of the subjects, insufficient specialized centers as well as financial resources contribute to this aspect. However, beyond these aspects, the fact that depression does not always involve physical pain, causes people not to associate their condition with a problem that needs to be detected and treated by a doctor. The difficulty in making these connections, essential in the decision to go to see or not to see a doctor, is given by the precarious health education regarding mental illness.

According to the research subjects, Romanians detect depression only in its advanced stages, the basis of this being, most often, the lack of information and health education, misperception of the real state, the desire to have total control over oneself, but also to fear of being judged, beyond the reasons related to the lack of financial resources and time.

Romanians’ interest in preventing, detecting, and treating depression will evolve in a positive way, in the view of research subjects, not only against the background of increasing importance given to this subject globally but also because Romanians are becoming more involved. For young people, the interest in mental health is shown by the choices they make: “(…) young people are more independent, they make choices emotionally, rather than financially (…)”, there are several tools to facilitate access to information, and technology is gaining ground for this purpose, such as increasing the number of users of lifestyle applications and physical exercise monitoring in our country. However, the subjects of the research consider that the increase in Romanians’ interest in preventing, detecting, and treating depression must be supported by effective campaigns carried out by medical units, NGOs, and the state.

In general, the interviewed subjects consider that the Romanian health system is stagnant because although there are premises and actions favorable to the evolution, there are also many gaps and deficiencies that cancel the positive aspects. However, those who consider that the Romanian health system is moving in a favorable direction, consider that this is due to the collective effort and the involvement of the civil society, rather than the reforms in the field. The subjects mention that for a positive and sustainable direction, Romania has a lot to recover, a lot of investments are needed in the field, and the approach of the system should really be directed towards prevention and not treatment, regardless of specialization.

Finally, research subjects describe mild depression as a common, at the same time complex condition that can be kept under control if detected in time, but which, unprevented or undetected in its early stages, negatively affects individuals’ quality of life.

### 3.2. The Results of the Semi-Structured In-Depth Individual Interview

Each topic in the interview guide involved a number of questions for the subjects in the sample. The data obtained were analyzed by the method of content analysis, the results being presented below for each topic of discussion. The first topic was to identify the level of knowledge of general perceptions and attitudes among participants in research on the new SARS-CoV-2 virus (COVID-19) and the evolution of the pandemic.

Research participants consider themselves “fairly well informed” about COVID-19 virus issues. All subjects stated that they had knowledge of the symptoms of the virus, its severity, social distancing measures, and rules for protection against the virus. “The media made sure that we all knew, whether we wanted to or not, things about this pandemic”, while three other subjects admit they are not sure how well they know about the evolution of the pandemic, because “I don’t know how accurate and complete the media information is.” The answers of the participants to this question reveal not only their level of knowledge (average to high) of aspects related to the new virus but also the importance of the media in informing them.

Of all the interviewees, five (41.6% of the participants) found out about the onset of the coronavirus pandemic through television. Six participants said they found out about the outbreak of the pandemic in the online press, nine of the subjects found out through Social Media, while six participants gave more general answers: “from the media” and “from the Internet.”

The general reactions among the participants were divided between feelings of fear (“I was afraid”, “Fear”, etc.), anxiety (“I panicked a little”, “I was agitated”, “I became anxious”), “I started thinking about what’s worse”, “I had a kind of shock”) and ignorance (“I wasn’t really interested”, “I didn’t pay much attention”, “Somehow I ignored the subject, at the beginning”).

For more than half of the participants in the research, a correct source of information on COVID-19 is represented by health professionals, such as specialists doctors, family doctors, health authorities and authorities involved in managing the COVID-19 crisis (Government, Ministry of Internal Affairs, Ministry of Health, Public Health Department, local authorities), while for five of the participants, the media represents a correct source of information about the new virus. However, of the five participants who nominated this way of information, more than half clearly mention the need for the media to verify the information (“press with verified sources”, “verified sources on the Internet”, “online media, with source verification”).

In order to accurately determine participants’ perceptions, attitudes, and potential changes regarding their mental health concerns, they were asked to outline their experience with the new virus. The most used terms in describing the experiences of the participants were: “unpleasant”, “scary”, “sequels”, “fear”, “horror”, “stress”, “insecurity”, “loneliness”. It should be noted that the manifestation of feelings of fear, insecurity, and anxiety occurred not only among participants who at one time were COVID-19 patients, being infected with this virus but also among those who were declared direct contacts of a COVID-19 patient at one point: “I was in confinement for 2 weeks because of a friend. It was a difficult experience, especially mentally”; “It mentally affected me, because I was and still I am afraid of getting infected or infecting others. I’ve lost touch with the world, I don’t think I’ll ever be the same again”; “A struggle with loneliness, with the fear of getting sick or infecting someone”). Participants also mentioned the negative effects of the COVID-19 experience, beyond the impact on their mental health: “(…) I lost a lot: money, job, social life, health”, “(…) It has completely changed my lifestyle”, etc.

The participants in this research were asked to describe, in a word, their first thought about cases of infection with the new coronavirus. All associated words (see Table 2) reflect the negativity of the phenomenon.

The restrictive measures implemented throughout the evolution of the COVID-19 pandemic, as well as the protection measures imposed to limit the spread of the virus, have generated behavioral and lifestyle changes for the research participants. Thus, beyond the compliance with wearing the mask, most of the subjects stated that they limited their travels, and that they were isolated as much as possible, this meaning a limitation of the social interaction (“(…) I stayed away from people”, “I avoided contact with people”, ”I gave up physical socialization”, “(…) I did not visit my family”, etc.). Many participants also mentioned changes in their buying behavior, some of them switching to online shopping, while others changed their buying behavior in the sense of decreasing their buying frequency (for example: “… I went shopping less often”, “Rarer and more consistent shopping”). Moreover, some of the participants stated that they bought and self-administered preventive medicine, without being infected with the virus or experiencing any other disease (“I took preventive medicine”). Some of the subjects mentioned changes in their behavior regarding transport, giving up traveling by public transport (“(…) I did not go by bus anymore”). Giving up family visits, eliminating socialization, giving up traveling, canceling meeting friends or physical contact with peers are among the most common measures that have had the greatest impact on the well-being of people participating in research.

Participants perceive life during the pandemic as difficult, uncertain, frustrating, and “more complicated than usual”. Some subjects perceive life being more costly as a result of declining incomes, incurring expenses from the purchase of protective equipment, such as masks, gloves, or disinfectants, and the abandonment of public transportation in favor of personal cars or alternative means. One of the subjects perceives life during the pandemic as if “struck by anxiety and apathy”. Anxiety is a common phenomenon among subjects’ perceptions of life in the pandemic context, including feelings of panic, frustration, sadness, and fear.

Among the greatest concerns of the research participants about the COVID-19 pandemic are fear of getting sick the first time or getting sick again (in the case of those who have already been infected with the virus), fear of spreading the virus, or even fear of death of loved ones. At the same time, a large part of the subjects are afraid of uncertainty, one of the subjects stating: “I am afraid that we will not return to normal, that we will live only in uncertainty.” Another fear of the participants is related to the sequelae and emotional traumas generated by this pandemic (“I am afraid that we will be left with traumas, that everything that happens now will become normal”), or the adaptation problems (e.g., “I’m afraid I’ll have problems with employment because of the online school”, “I’ll have problems in adapting”, “(…) I won’t be able to communicate as before”, “We will be afraid of people and we will no longer be united”, “I am afraid we will not behave human anymore” etc.). While some participants fear potential inefficiencies of COVID-19 vaccination, there are also participants who fear the health system’s inability to manage these pandemics effectively (“I’m afraid hospitals will collapse”). Beyond these aspects, the anxiety and fears of the participants are also related to the financial aspects, such as the fear that they will suffer financially, the fear that they will experience major social and economic–financial crises, etc. However, fears related to the resumption of social activities are among the most common, the participants being afraid of loneliness, of the inability to resume social activities (“(…) we will forget what fun means”, “(…) we will live in paranoia and we will be afraid of each other”, “(…) no one will visit me anymore.”

21 of the 36 subjects in the study said that the COVID-19 virus affected their mental health. The rest of the participants, although they did not strongly state that this virus has affected their mental well-being, tended to believe that the COVID-19 pandemic has had some effect on them as well. Of those convinced that the pandemic has affected their mental well-being, most say they get sad faster, fear easier and more, feel a higher level of stress, and even become frustrated, nervous, or irritable much faster than during pre-pandemic. Those who are not convinced that their changed attitude and behavior are related to their mental health state that they lose patience faster, that they perceive the negativity of things more quickly, and that they are more disorganized and lack motivation. Three of the subjects said, “I don’t know, I think we’re all more anxious, nervous, sad, confused and irritated.”

Three participants are not convinced of the influence of the pandemic on temperament, feelings, or emotion. Although they deny the influence, they worry faster and think more about what might be wrong. The rest of the research participants are aware of the pandemic effects on their emotions, feelings, and temperament, stating that the virus had clear effects in terms of intensification of feelings and emotions (“(…) stronger emotions, mixed emotions”, “(…) I became more emotional”, “(…) fear has taken more over my psyche”), increased anger and feelings of frustration, often leading to the transition to temperament more violent, more aggressive than initially: “(…) more recalcitrant than before”, “(…) I get upset faster, I get angry faster”, intensifying negative feelings (“(…) I’m more negative, colder with those around”). These changes were mainly determined by the restrictive measures of movement and social distancing, as well as by the financial, economic, medical uncertainties, etc. generated by the pandemic context.

Combating the fears and anxiety caused by the COVID-19 pandemic is important for the mental well-being of the population. Asked about the methods used to combat anxiety and fear related to this pandemic, among the most nominated were: music for relaxation (“I take my time (…) listening to music”, “(…) I listen to music”, “(…) listening to relaxing music”), communication through technology (“communicating online with friends”, “talking on the phone with parents”, “communicating online with loved ones”), using technology tools for various purposes related to mental or physical well-being (“I use my phone a lot for various purposes”, “I monitor my condition with various mobile applications”, “I use applications for meditation”, “I monitor my condition with profile applications”, “I use applications that measure my sleep, nutrition, stress level”, “I scroll through positive information, texts and videos”). Notable is the statement of a subject who mentions that in order to combat anxiety and fear related to the pandemic, he/she avoids television. Thus, communication with loved ones through technology and the use of technology tools for both medical purposes (physical activity monitoring, meditation) for information and entertainment (videos, music, relaxation games) reflects the importance of these tools in self-care management of anxiety and mild depressive symptoms.

In the context of the COVID-19 pandemic, the majority (30 participants) benefited from medical services. Of these, 12 people accessed medical services physically, by hospitalization, or by calling the unique emergency number 112, as a result of COVID-19 infection and presenting symptoms in aggravating forms. Most of those who accessed medical services during this period benefited from remote medical services through technology (telemedicine, communication with the family doctor through technology platforms: e-mail, WhatsApp, and even Facebook, or through applications such as SanoPass). These services were mainly health monitoring services. Some participants did not receive specific medical services. For some subjects, access to medical services proved to be a stress factor due to the overcrowding of health care providers on the one hand (“(…) It was difficult, the hospitals were full, I waited a long time”, “(…) It was very stressful, especially because the situation in the hospitals is deplorable and it is known what the outbreaks of infection are in the hospitals”). For other participants in the research, a stress factor in accessing medical services was technology itself, through the impediments related to technical issues: “It was stressful, because it was difficult to establish contact.” However, for some of the participants in the research, accessing remote medical services generated less stress than accessing physical health services (“It was not a stressor”, “It was not very stressful”, “It was not at as stressful as the visit to the office”, ”(…) it was less stressful than the physical visit to the hospitals”). Thus, from the discussions with the research participants, it is concluded that remote medical services, through technology tools, are useful alternatives in pandemic contexts and can increase the accessibility of the population to health services, at the same time with a decrease of stress, as long as investments are made in ICT health infrastructure and there is a coherent plan for coordinating such services. The most commonly used methods for information and communication by research participants during the pandemic are listed in the table below (Table 3).

For information, most research participants nominated the online press as a means of information, followed by TV and Social Media. At the same time, for communication, the most used means are represented by Social Media tools (Facebook, WhatsApp, Instagram, etc.), followed by phone calls and video conferencing platforms such as Zoom, Skype, or Microsoft Teams.

In order to determine the methods of support and their accessibility for the population, especially for their mental well-being in the context of the COVID-19 pandemic, this sub-topic was addressed in the interview guide. Thus, beyond the support from doctors, especially family doctors, in terms of information, medical services provided, etc., participants identified support in employers and authorities who facilitated their work remotely and provided them with administrative facilities. (“Possibility to work from home”, “Postponement of rates”). In addition, a large number of research subjects mentioned technology as a form of support and assistance during this period, especially for communication, access to information, and work or education activities. It can be said that each individual has formed a mechanism to deal with the COVID-19 pandemic. Many of the participants in the research mentioned that in the context of the pandemic, their closeness to God, faith, spirituality, and prayer helped them manage the situation generated by the pandemic. Moreover, time spent at home with family, connecting with loved ones, and communicating peacefully with close ones are other ways in which research participants felt they could handle the pandemic situation. From this point of view, although social distancing is seen as a negative aspect of the population, for many of the participants in the research it was also a positive aspect: the recovery of time spent with the family, the comfort generated by the family.

Regarding the need for mental health programs and/or minimum measures to overcome anxiety, fear, stress, and mild depressive symptoms in the context of the pandemic, all participants considered it appropriate to set up a program to support the mental health needs of the population in these times.

Research participants believe that coherent strategies and programs are needed to promote the mental health of the population. Although there are initiatives from public figures, community leaders, or other volunteers, research participants highlight the need for an effective, well-structured, and functional national program for the mental well-being of the population. Some participants believe that there is a need to “launch campaigns to raise awareness of the dangers of a pandemic to our mental and physical health”, in addition to the much-needed investment in medical and ICT infrastructure to provide telemedicine services, especially for the elderly or those at risk, in a well-coordinated and efficient manner in our country. Some participants consider the creation of a web or mobile application “to address the problems of the population (especially mental ones) in times of crisis or pandemic” as an effective way to inform, raise awareness and manage possible future pandemic contexts, emphasizing at the same time, the need to invest in technology-assisted health programs and mental health services. Another research topic considered appropriate is to have courses and training in schools and local communities to train and test the reactions of the population in crisis contexts. Generally, depression and anxiety prevention campaigns, mental health promotion campaigns, funding the mental health services, implementation of remote mental health services through technology, and investments in the creation of programs, strategies, and platforms for the management of pandemic contexts, implicitly mental health issues in those contexts are considered necessary and effective by research participants to be prepared for potential future pandemic contexts.

### 3.3. Survey Results

Quantitative research outlined the answers of 514 subjects and helped the authors find new insights regarding the importance given to mental health. The survey revealed information about factors that stimulate mental health services about the level of information on the symptoms of depression and the budget allocated for prevention.

**Figure 1:**
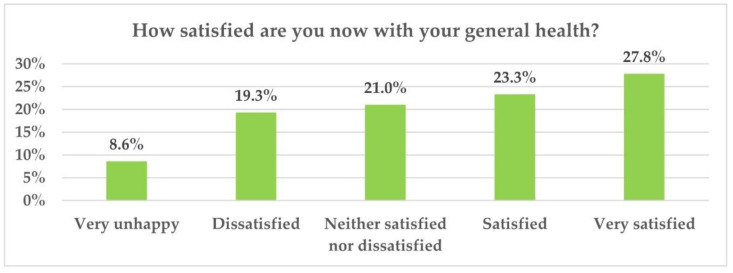
Degree of satisfaction with own state of health (N = 514).

To the question “Currently, how satisfied are you with your overall health?”, 8.6% of all respondents indicated total dissatisfaction (Figure 1).

**Figure 2:**
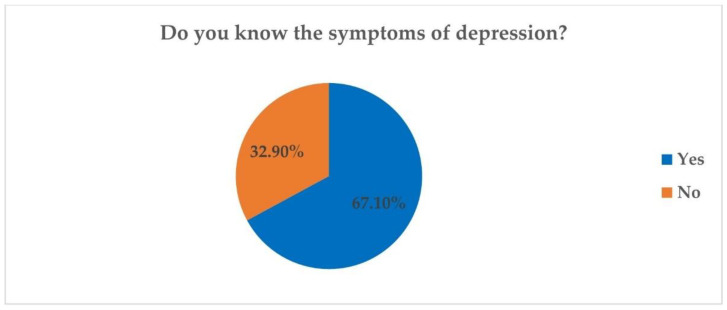
Awareness of depressive symptoms (N = 514).

Regarding the percentage of those who know the symptoms of depression at the sample level, 67.1% of the 514 members of the sample stated that they know the symptoms of depression, while 32.9% of the total respondents did not know the symptoms of this condition (Figure 2).

Of the 514 respondents to this question, 67.9% stated that they had never participated in a specialist assessment for their mental health, while 32.10% stated that they had assessed their mental health status by a specialist at least once. Regarding the last visit to the specialist for specialized assessments on mental health, of the 171 respondents who stated that they participated in specialized controls, 27.5% stated that these visits to the specialist took place more than two years ago, while 29.8% said that the last visit to the specialist took place in the last 6–12 months. 25.7% of respondents stated that their last visit to the specialist took place in the last two years, and 17% went to a specialist for their mental health in the last month.

With the help of inferential statistics, a hypothesis was verified regarding the amount allocated by Romanians, annually, for the prevention of depression (Table 4 and Table 5). At the time of the study, the cost of a specialized consultation in the mental health field, in Romania, was between 150 and 200 lei. The chosen value of 180 lei is a hypothesis set by the authors based on the average cost of an analysis package. Table 4 shows the average obtained at the sample level. Thus, the following statistical hypotheses were taken into account:

**H_0_.**  *The average annual amount allocated by Romanians for the prevention of depression is 180 lei (µ = 180 lei).*

**H_1_.**  *The average annual amount allocated by Romanians for the prevention of depression is different from 180 lei (µ ≠ 180 lei).*

Official exchange rate: 1 euro = 4.94 lei

**Table 4:** Descriptive statistics indicators at the sample level (N = 514).

	N	Mean	Std. Deviation	Std. Error Mean
*Approximately, how much do you spend on average (in lei) per year to prevent depression?*	514	212.6381	340.0616	14.9995

**Table 5:** T-Student test for hypothesis testing (N = 514).

	Test Value = 180					
	t	df	Sig. (Two-Tailed)	Mean Difference	95% Confidence Interval of the Difference	
					Lower	Upper
*Approximately, how much do you spend on average (in lei) per year to prevent depression?*	2.176	513	0.030	32.63813	3.1702	62.1061

At the level of the sample, the average annual amount allocated for the prevention of depression is 212.64 lei. To test the hypothesis, the Student’s t test was applied in the case of univariate analysis.

By applying the t-Student test in the case of univariate analysis, the value t_calc_ = 2.176 was obtained, not belonging to the interval [−1.96; +1.96]; therefore, the alternative hypothesis, H_1_, will be accepted. The decision is also confirmed by the comparison of the level of significance Sig. (2-tailed) = 0.03, with the theoretically considered significance level of 0.05. Since the level of significance Sig. (2-tailed) is lower than the theoretical level of significance (0.03 < 0.05), it is guaranteed, for 95% probability, that the average annual amount allocated for the prevention of depression by the adult population is different from 180 lei.

According to the information obtained at the sample level, it can be concluded that the major population in Romania allocates, annually, more than 180 lei for the prevention of depression.

At the same time, a hypothesis regarding the connection between the level of education and the agreement/disagreement regarding the statement “Depression is a preventable condition” was tested.

**H₀.**  *There is no connection between the level of education of the adult population and the statement “Depression is a preventable condition.”*

**H₁.**  *There is a connection between the level of education of the adult population and the statement “Depression is a preventable condition.”*

In this sense, the variable regarding the level of education has been transformed, being generated into two groups: respondents whose last school graduated is the general school/less than 10 classes/vocational school/high school and respondents who graduated post-high school/faculty/postgraduate studies.

At the level of the sample, 272 respondents belonging to the group with high school as the highest form of graduate education answered, with an average score of 3.26 points answered by this group, on the scale where 1 = “Total disagreement” and 5 = “Total agreement”. 242 respondents form the group whose studies are higher than high school, and the average score obtained for this group was 4.14 points on the same scale (1 = “Total disagreement”, 5 = “Total agreement”) (Table 6). Differences can also be observed in the standard deviations for the two groups. To test the significance of this difference from a statistical perspective, the t-Student test was used in the case of bivariate analysis, starting from the previously mentioned hypothesis (H_0_: μ1 = μ2 and H_1_: μ1 ≠ μ2).

In Levene’s test of equality of variances (Table 7), a level of significance Sig = 0.00 was obtained, positioned below the threshold of 0.05, so it can be guaranteed that, for a probability of 95%, the variances of two groups are different. Therefore, the results on the second row (Equal variances not assumed) will be read. A value of t_calc_ = −9.622 was obtained, which does not belong to the range [−1.96; +1.96], which leads to the rejection of the null hypothesis, H_0,_ and the acceptance of the alternative, H_1_, i.e., for a probability of 95% can be guaranteed the existence of a link between the level of education and the agreement/disagreement regarding the statement “Depression it is a preventable condition”. Therefore we can guarantee, under the established probability conditions, that there are differences between the two groups in terms of the total population. Significance Level Sig. (2-tailed), which is equal to 0.00, being below α = 0.05, confirms the decision.

Another hypothesis aimed at verifying the connection between the occupation of the inhabitants of Brașov county and the assessments regarding the importance of technology in informing about diseases such as depression. In this sense, we started with the following statistical hypotheses:

**H_0_.**  *There is no connection between the occupation of the people of Brasov and the assessments regarding the importance of technology in informing about conditions such as depression.*

**H_1_.**  *There is a link between the occupation of the people of Brasov and the assessments regarding the importance of technology in informing about conditions such as depression.*

Following the analysis of the data in Table 8, differences can be observed between the averages at the level of the groups. For example, at the level of the group of retirees, the average is higher than the averages of the other groups. The lowest average of the assessments is observed at the level of the group of pensioners—2.13 points on the scale used (1 = “Not at all important”, and 5 = “Very important”), while the highest average belongs to the group of operative workers—4.24 points on the same scale. To test the statistical significance of these differences, a test was performed for the differences between the group variances, the ANOVA test, and a statistical test for the variance equality hypothesis.

*To what extent do you think technology is important for information on conditions such as depression?*

It may be observed that the F_calc_ value is equal to 2.924 (Table 9). This will be compared with a critical value in the Fisher’s distribution table, chosen based on the significance level α = 0.05, df1 = 6, and df2 = 507. This value will be calculated using the FINV function in Excel. F_calc_ = 2.924 is greater than F_0.05; 6; 507_ = 2.116, which means that the alternative hypothesis, H_1_, will be accepted, meaning the possibility of guaranteeing, with a probability of 95%, that the variances regarding the assessments of the importance of technology in mental health information are different for occupational groups. The same conclusion is reached according to Asymp Sig = 0.008, which is less than α = 0.05. The testing of the differences between the means, in order to highlight the existence of the connection between the two variables, will be performed on the basis of the analysis table of the variances (Table 10).

By comparing the value of F_calc_ with the value of F_0.05; 6; 507_, it can be seen that:

F_calc_ = 37.192 (Table 10) is higher than F_0.05; 6; 507_ = 2.116, so that the alternative hypothesis H_1_, according to which there is a link between occupation and assessments of the importance of technology in mental health information, will be accepted instead of the null hypothesis, H_0_.

In Table 11, with the sign * are marked all the pairs of averages that are, for a level of significance α = 0.05, significantly different. Therefore, observing the confidence intervals (95% Confidence Interval in Table 11), the following conclusions can be drawn: for confidence intervals containing the value zero, the null hypothesis H_0_ is accepted, according to which it cannot be guaranteed with a 95% probability that the averages of the respective groups are different, while for the rest of the ranges, the alternative hypothesis H_1_ will be accepted, i.e., it can be guaranteed, under the same probability conditions, that the averages of the respective groups are different.

The same decisions can be made according to the level of significance of Sig., this being lower than 0.05 in the 14 cases and higher than 0.05 in the other cases.

## 4. Discussion

Mental health is an extremely sensitive issue for the period that humanity is currently going through; the assessment of the attitudes regarding mental health has led to the use of three distinct research methods, the results obtained being similar to those obtained by other researchers [32,35,50,51,52,53]. Depression not only means sadness, irritability, insomnia, or melancholy, but it can mean chronicity, aggravated heart disease, and so on. The specialists included in the study state that the prevention of depression requires balance, whether we are talking about lifestyle, social integration, or crisis management. At the same time, in their view, prevention involves informing and expressing health care through regular medical evaluations. For this purpose, the detection and treatment of depression involve, first and foremost, effort and willingness from the population. Evaluation by clinical interview, based on scalediagnosis, found that all specialized tests to detect depression and then building a treatment scheme, would not be possible without the sincerity of patients, their information, discipline, and consistency in activities. At the same time, it would not be possible to prevent, detect or treat depression without providing patients with the information and without providing doctors with the tools necessary for this purpose.

**Figure 3:**
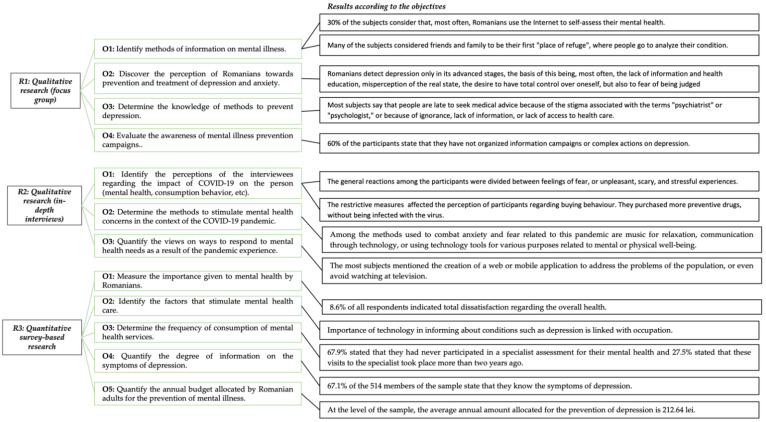
The overall structure of the research in terms of objectives and results.

Therefore, the results of this study fit the context. Moreover, the established objectives were achieved and the unexpected results, presented carefully in the context (Figure 3) may now contribute with a real value to the scientific world.

The first research conducted started with four objectives, all of them achieved, which, surprisingly, generated higher value than expected. The first objective was conducted to the identification of many methods of information on mental illness. These include the Internet, face-to-face communication, blog, Social Networks, websites, outdoor banners, family doctors’ networks, specialized forums, flyers, and social media, or family members as sources. These results led the authors to a verdict on the perception of Romanians towards the prevention and treatment of depression and anxiety (the second objective). It is strongly related to the stigma associated with the terms “psychiatrist” or “psychologist”, which conduct to late seek medical advice [16]. This fact would be avoided if the stigmatization of mental and behavioral disorders [13,15] through all existing means of communication would be diminished. At that moment, the patients will stop to ignore the assessment of mental health with the help of specialists and will start to detect depression in its early stages. All these will contribute to a stronger resilience [20,21,22,23,24,25].

The results obtained regarding the weaknesses of the Romanian health system in this field, the lack of staff, lack of infrastructure, lack of clear protocols, untrained family doctors in terms of mental and behavioral disorders, lack of national campaigns on these diseases, lack of investment in the field, lack of prevention services, corruption, lack of health education, overcrowded hospitals, excessive bureaucracy, disinterest, and weak communication, cover the third objective related to the knowledge of methods to prevent depression. It logically drew up the evaluation of the awareness of mental illness prevention (the fourth objective) as being very low. Moreover, it shows the strong necessity for complex actions on depression prevention with high-quality afferent campaigns and public policies.

Therefore, modernization in the health field is urgently needed both in terms of public health services and the health system. However, many factors contribute to the achievement of this goal, including financial resources, political stability, and legislative resources. Some of the shortcomings of this field in our country can be ameliorated by the transition to prevention services, especially in terms of mild mental illness. It is desirable that prevention and preventive services in mental health become a priority. Considering that prevention is based on correct information about the population, correct and effective education of the population, and communication between stakeholders (specialists and population), new technologies and digitization can play an important role in this issue.

The second research generated satisfying results to declare objectives achievement considered relevant for COVID-19 pandemic studies [43,56,57]. The perceptions of the interviews regarding the impact of the pandemic were identified (O_2_), the participants declaring the feelings of fear increasing or unpleasant experiences intensifying, and even behavioral changes in buying frequency (less often), or more often in the case of purchasing preventive drugs. Among the methods used to combat anxiety and fear related to this pandemic are music for relaxation, communication through technology, or using technology tools for various purposes related to mental or physical well-being [6,9]. All these options achieved the third objective. The O_3_ was achieved by quantifying the views on ways to respond to mental health needs because of the pandemic experience. In these terms, most of the subjects mentioned the creation of a web or mobile application to address the problems of the population, or even avoid watching television.

Simultaneously with the pandemic crisis, the world economy is preparing to enter a new economic crisis. In Romania, as in many important countries, we are currently witnessing a sharp decline in purchasing power due to the high rate of inflation which, fortunately, is not doubled by a significant increase in the unemployment rate. Unfortunately, the research conducted could not include the influences that the economic crisis could have on the well-being of Romanian citizens because, at the time of elaboration, this issue was not anticipated with certainty. However, it must be considered because the consequences can certainly affect the well-being and, implicitly, the mental health of citizens. In the social sphere and the health sphere, public mental health policies aim at generating changes for the better in consumer behavior. In carrying out programs related to mental health, the essential points are related to the identification and effective segmentation of the target audience, the identification of the benefits generated by behavioral change, and the size of the program (local, regional, national, global). Health marketing, through its specific strategies and programs, together with the integration of digital tools in the field to a greater extent, might contribute to managing the challenges of depression prevention in Romania, especially considering the role of marketing in the field.

For this reason, all the valuable information obtained through qualitative research led to the quantitative research results, enriching studies in the field [8,19]. All the objectives were achieved, the authors presented the big picture of the importance given to mental health by Romanians, the factors that stimulate the mental health care, the low frequency of consumption of mental health services, the low level of information on the symptoms of depression, and the low budget allocated by Romanian adults for the prevention of mental illness.

Notwithstanding, health, by its interdisciplinarity and its contribution to economic and social development, plays a role in maintaining health and encouraging healthy consumer behavior.

Of course, addressing and implementing programs to cover the challenges of depression require knowledge and adaptability to models of health systems and the state in which they are found. The gaps regarding the organization and financing of the Romanian health system must be considered not only for the national strategy on mental health but also for the definition and creation of specific, sustainable marketing programs. In Romania, the context of digitalization in health is still fragile, for financial reasons (budget allocations), due to poor infrastructure, but also human resource problems. Therefore, implementing solutions in strategies, marketing programs, and public policies in the field of mental health, involves primarily the preparation of the system from a legal, financial, but also educational perspective. Therefore, beyond the ICT infrastructure, the medical and health personnel, the education of the population, its digital skills, and the openness to the adoption of new technologies are becoming increasingly important in defining public policies based on digital tools for the prevention of conditions such as depression.

## 5. Conclusions

The authors’ approach is not a medical one; it is not based on experiments and observations but rather focuses on the correct identification of behaviors in order to develop effective public health policies. The proposals aim at involving the executive branch, especially through the Ministry of Health, as well as academia. Being a health problem with a massive negative impact on the economy, and the well-being of the population, the collaboration between the specialists of the academic environment and the Ministry of Health becomes obviously necessary. On the one hand, the academic environment must develop and perpetuate research to detect behavioral changes and worsening mental health problems, both among specialists and the population, and on the other hand, the Ministry of Health must work with the academic and economic environment in order to attract the necessary financial resources for the development of public health policies that lead to the well-being of the population with positive consequences on economic and social life.

The main limitation of the research is the method of the group interview, where there was a possibility that some of the participants may have been influenced by the answers of the other subjects. Moreover, the researcher was not sure that the information provided by the participants was completely honest, given the high degree of sensitivity of the topic chosen for this research. Another limitation of qualitative research refers to the possibility for the researcher to perceive differently the meaning of the words expressed by the participants, especially in the topics where projective methods were used. The third limitation of the qualitative focus group research undertaken refers to the large amount of information extracted from the two group interviews, which can lead to the loss of sight of some essential aspects of the discussions. Last but not least, a limitation of qualitative research is the impossibility of extrapolating the results obtained.

Future research direction aims at qualitative research to be developed and marketing experiments but also quantitative research based on representative samples and probabilistic research methods to monitor the evolution of mental health of the population while promoting the effectiveness of public health programs promoted.

## Figures and Tables

**Table 1:** Prevalence of mental disorders in 2019 (of which: depressive/anxiety disorders).

Location	Cause	Number	Percent	Rate
Global	Mental disorders	970,070,243.21	13.04	12,537.31
Western Europe		67,021,711.29	16.21	15,361.12
Central Europe		12,877,710.52	11.79	11,274.12
Romania		2,197,434.97	11.89	11,422.92
Global	Depressive disorders	279,606,278.74	3.76	3613.67
Western Europe		20,011,087.03	4.84	4586.47
Central Europe		3,770,271.81	3.45	3300.78
Romania		647,908.86	3.51	3368.02
Global	Anxiety disorders	301,390,390.85	4.05	3895.21
Western Europe		26,057,799.71	6.30	5972.35
Central Europe		4,231,801.64	3.87	3704.84
Romania		713,811.81	3.86	3710.61

Data extracted from Global Health Data Exchange (GHDx).

**Table 2:** Words associated with COVID-19 cases (N = 36).

No.	Associated Words	Frequency
1	Fear	15 (41.6%)
2	Sadness	6 (16.6%)
3	Panic	3 (8.3%)
4	Frustration	3 (8.3%)
5	Helplessness	3 (8.3%)
6	Resignation	3 (8.3%)
7	Anxiety	3 (8.3%)

**Table 3:** Methods and means of information and communication during the COVID-19 pandemic (N = 36).

Methods and Means of Information	Methods and Means of Communication
Online Media—66.6%	Social Media—91.6%
TV—50%	Phone—58.3%
Social Media—50%	Online video communication platforms—33.3%
Websites—33.3%	E-mail—25%
Friends—25%	

**Table 6:** Group statistics (N = 514).

	Level of Education	N	Mean	Std. Deviation	Std. Error Mean
*“Depression is a preventable condition”*	Primary and secondary education	272	3.2610	1.14690	0.06954
	Higher education	242	4.1405	0.92279	0.05932

**Table 7:** T-Student test results for differences in averages (N = 514).

		Levene’s Test for Equality of Variance		T Test for Equality of Means						
		F	Sig.	t	df	Sig. (2-Tailed)	Mean Difference	Std. Error Difference	95% Confidence Interval of the Difference	
									Lower	Upper
*“Depression is a preventable condition”*	Equal variance assumed	15.687	0.000	−9.502	512	0.000	−0.87947	0.09256	−1.06130	−0.69763
	Equal variance not assumed			−9.622	507.008	0.000	−0.87947	0.09140	−1.05904	−0.69989

**Table 8:** Descriptive group statistics (N = 514).

	N	Mean	Std. Deviation	Std. Error Mean	95% Confidence Interval of the Difference		Minimum	Maximum
					Lower	Upper		
Manager/Employer	50	3.8800	1.09991	0.15555	3.5674	4.1926	1.00	5.00
Professional/Tertiary education employee	116	4.1293	0.88985	0.08262	3.9657	4.2930	2.00	5.00
Pupil/Student	159	3.5094	1.08422	0.08598	3.3396	3.6793	1.00	5.00
Operator	58	4.2414	0.84418	0.11085	4.0194	4.4633	1.00	5.00
Unemployed	31	2.8065	1.01388	0.18210	2.4346	3.1783	1.00	5.00
Pensioner	52	2.1346	1.13809	0.15782	1.8178	2.4515	1.00	5.00
Housewife	48	2.6250	1.10367	0.15930	2.3045	2.9455	1.00	5.00
Total	514	3.5039	1.22075	0.05385	3.3981	3.6097	1.00	5.00

**Table 9:** Levene test for differences in group variances (N = 514).

Levene Statistic	df1	df2	Sig.
2.924	6	507	0.008

**Table 10:** Variance analysis table (N = 514).

	Sum of Squares	df	Mean Square	F	Sig.
Between groups	233.649	6	38.941	37.192	0.000
Within Groups	530.843	507	1.047		
Total	764.492	513			

**Table 11:** Tamhane test for the hypothesis of variance inequality (N = 514).

(I) Current Occupation	(J) Current Occupation	Mean Difference (I-J)	Std. Error	Sig.	95% Confidence Interval	
					Lower Bound	Upper Bound
Manager/Employer	Professional/Tertiary education employee	−0.24931	0.17613	0.975	−0.8011	0.3025
	Pupil/Student	0.37057	0.17773	0.578	−0.1855	0.9266
	Operator	−0.36138	0.19100	0.737	−0.9569	0.2341
	Unemployed	1.07355 *	0.23949	0.001	0.3194	1.8277
	Pensioner	1.74538 *	0.22160	0.000	1.0563	2.4345
	Housewife	1.25500 *	0.22265	0.000	0.5618	1.9482
Professional/Tertiary education employee	Manager/Employer	0.24931	0.17613	0.975	−0.3025	0.8011
	Pupil/Student	0.61988 *	0.11925	0.000	0.2550	0.9847
	Operator	−0.11207	0.13825	1.000	−0.5402	0.3160
	Unemployed	1.32286 *	0.19997	0.000	0.6790	1.9667
	Pensioner	1.99469 *	0.17814	0.000	1.4371	2.5523
	Housewife	1.50431 *	0.17945	0.000	0.9410	2.0676
Pupil/Student	Manager/Employer	−0.37057	0.17773	0.578	−0.9266	0.1855
	Professional/Tertiary education employee	−0.61988 *	0.11925	0.000	−0.9847	−0.2550
	Operator	−0.73195 *	0.14029	0.000	−1.1656	−0.2982
	Unemployed	0.70298 *	0.20138	0.023	0.0558	1.3502
	Pensioner	1.37482 *	0.17973	0.000	0.8130	1.9366
	Housewife	0.88443 *	0.18103	0.000	0.3169	1.4519
Operator	Manager/Employer	0.36138	0.19100	0.737	−0.2341	0.9569
	Professional/Tertiary education employee	0.11207	0.13825	1.000	−0.3160	0.5402
	Pupil/Student	0.73195 *	0.14029	0.000	0.2982	1.1656
	Unemployed	1.43493 *	0.21318	0.000	0.7561	2.1138
	Pensioner	2.10676 *	0.19286	0.000	1.5059	2.7076
	Housewife	1.61638 *	0.19407	0.000	1.0105	2.2223
Unemployed	Manager/Employer	−1.07355 *	0.23949	0.001	−1.8277	−0.3194
	Professional/Tertiary education employee	−1.32286 *	0.19997	0.000	−1.9667	−0.6790
	Pupil/Student	−0.70298 *	0.20138	0.023	−1.3502	−0.0558
	Operator	−1.43493 *	0.21318	0.000	−2.1138	−0.7561
	Pensioner	0.67184	0.24097	0.134	−0.0864	1.4300
	Housewife	0.18145	0.24194	1.000	−0.5802	0.9431
Pensioner	Manager/Employer	−1.74538 *	0.22160	0.000	−2.4345	−1.0563
	Professional/Tertiary education employee	−1.99469 *	0.17814	0.000	−2.5523	−1.4371
	Pupil/Student	−1.37482 *	0.17973	0.000	−1.9366	−0.8130
	Operator	−2.10676 *	0.19286	0.000	−2.7076	−1.5059
	Unemployed	−0.67184	0.24097	0.134	−1.4300	0.0864
	Housewife	−0.49038	0.22424	0.485	−1.1881	0.2074
Housewife	Manager/Employer	−10.25500 *	0.22265	0.000	−1.9482	−0.5618
	Professional/Tertiary education employee	−1.50431 *	0.17945	0.000	−2.0676	−0.9410
	Pupil/Student	−0.88443 *	0.18103	0.000	−1.4519	−0.3169
	Operator	−1.61638 *	0.19407	0.000	−2.2223	−1.0105
	Unemployed	−0.18145	0.24194	1.000	−0.9431	0.5802
	Pensioner	0.49038	0.22424	0.485	−0.2074	1.1881

* The difference between the averages is significant at the level of 0.05.

## Data Availability

The data presented in this study are available online at shorturl.at/lwHK5 (accessed on 18 April 2022).
